# The prevalence of hypercalciuria in girl kids with over active bladder

**DOI:** 10.12861/jrip.2015.25

**Published:** 2015-11-29

**Authors:** Parsa Yousefichaijan, Mohammad Rafiei, Atefeh Aziminejad, Abdolghader Pakniyat

**Affiliations:** ^1^Department of Pediatric Nephrology, Arak University of Medical Sciences, Arak, Iran; ^2^Department of Biostatistics and Epidemiology, Arak University of Medical Sciences, Arak, Iran; ^3^Student Research Committee, Department of Emergency Medicine, Arak University of Medical Sciences, Arak, Iran

**Keywords:** Overactive bladder, Hypercalciuria, Children

## Abstract

**Introduction:** Hypercalciuria may be a sign of over active bladder, these symptoms may be treated if we get rid of hypercalciuria.

**Objectives:** This paper is intended to get to the prevalence of hypercalciuria in girl kids with over active bladder.

**Patients and Methods:** All patients with index of over active urinary bladder who admitted to Amir-Kabir hospital of Arak and children who have admitted without any particular disease just for a laboratory check were studied simultaneously. Urine sample was taken from both groups.

**Results:** The prevalence of hypercalciuria in the group with normal urinary bladder was 22.6% and in the case group was 42.9%. Hypercalciuria was reported in 30 (22.6%) children of control group and 57 (42.9%) children of case group. Based on chi-square test, hypercalciuria distribution between the two groups is not homogeneous (P = 0.001). Mean calcium to creatinine ratios were 2.384330 ± 0.55694 (mg/mg) and 2.186552 ± 0.56714 (mg/mg) for control and case groups respectively and no significant difference is observed between the two groups (P = 0.976). Based on logistic regression test, there is a significant relationship between both case and control groups and the occurrence of hypercalciuria with 2.58 times more than control group observed in case group.

**Conclusion:** Based on the high prevalence of hypercalciuria in case group, examination and treatment of hypercalciuria in patients with over active bladder may be effective.

**Implication for health policy/practice/research/medical education:**

Hypercalciuria may be a sign of over active bladder, hence examination and treatment of hypercalciuria in patients with over active bladder may be effective.

## Introduction

Over active urinary bladder is defined as urinary urgency without any specific pathogen or metabolic disease, may lead to incontinence, frequency or nocturia (1).

Twenty-five percent of children with nocturia have signs of over active urinary bladder. Many of them say that they do not feel urination until urinary incontinence. A history of urinary tract infection (UTI) is common among girls, however, urinary incontinence may continue long after urinary infection. It is not still clear whether voiding dysfunction is cause or result of UTI. In their voiding cystourethrogram (VCUG), sometimes dilatation of the ureter, narrowing of the bladder neck, or hypertrophied bladder wall may be observed (2).

Voiding every 1.5 or 2 hours is primary treatment. Constipation and UTI treatment, biofeedback or Kegel exercises are other useful treatments that may decrease bladder neck muscles contraction. Treatment with cholinergic (oxybutynin chloride, hyoscyamaine, tolterodine) and adrenergic α blockers (trazosin, doxazosine) may lead to the bladder relaxation (3,4).

Hypercalciuria is defined as more than 4 mg/kg calcium in 24-hour-urine or random calcium to creatinine ratio of more than 0.2 (5).

## Objectives

Based on the fact that hypercalciuria may be a sign of over active bladder, these symptoms may be treated if we get rid of hypercalciuria if present in patients. This paper is intended to get to the prevalence of hypercalciuria in girl kids with over active bladder.

## Patients and Methods

In a cross-sectional study, case group consisted of 131 girls above 5 years with over active bladder and control group included 131 girl who had referred for a laboratory test without any disease. Control patients were selected from those who admitted for laboratory test with normal clinical status. Calcium and creatinine urinary amount were measured. Patients with urinary infection were excluded and calcium to creatinine ratio was considered as random calcium to creatinine ratio of more than 0.2 (5).

Before this study took place, the procedure was explained to kids and their parents and the urine sampled for disease diagnosis were used and no additional cost imposed to patients. The study was performed under the supervision of research and ethics committee of university and hospital and information of patients were private.

### Ethical issues

1) The research followed the tenets of the Declaration of Helsinki; 2) informed consent was obtained, and they were free to leave the study at any time; and 3) the research was approved by the ethical committee of Arak University of Medical Sciences.

### Statistical analysis

Data were analyzed by SPSS 16 software. Descriptive statistics including frequency, percentage, mean, standard deviation and analytic statistics including logistic regression, odd ratio (OR) and chi-square test were utilized. P value with rate of less than 0.05 was considered as significant level.

## Results

The prevalence of hypercalciuria were 22.6% in control group and 42.9% in case group. Thirty patients (22.6%) of control group and 57 patients (42.9%) of case group had hypercalciuria. Based on chi-square test, the distribution of hypercalciuria is not homogenous in the two groups (*P *= 0.001) (Table 1).

**Table 1:** The frequency of hypercalciuria in girls (5-12 years) with and without over active bladder referring to Amir Kabir hospital during 2012-2013

		**Normal**	**Abnormal**	**Total**
Case	No.‏ (%)	103 (77.4)	30 (22.6)	133(100)
Control	No.‏ (%)	76 (57.1)	57 (42.69)	133(100)
Total	No.‏ (%)	179 (67.3)	87 (32.7)	266(100)

Mean calcium to creatinine ratios were 2.384330 ± 0.55694 mg/mg) and 2.186552 ± 0.56714 (mg/mg) for control and case groups respectively and no significant difference is observed between the two groups (*P* = 0.976) (Table 2).

**Table 2:** Mean and standard deviation of hypercalciuria, creatinine and calcium to creatinine ratio in girls (5-12 years) with and without over active bladder referring to our hospital during 2012-2013

		**No.**	**Mean**	**SD**	***P*** ** Value**
Calcium (mg/dl)	Control	133	20.172	26.55	0.635
	Case	133	21.78	28.56	
Creatinine (mg/dl)	Control	133	137.35	127.23	0.000
	Case	133	84.65	73.46	
Calcium/Creatinine (mg/mg)	Control	133	0.55	2.38	0.97
	Case	133	0.56	2.18	

According to logistic regression test, there is a significant relationship between both case and control groups and the occurrence of hypercalciuria with 2.58 times more than control group observed in case group. (OR = 2.58 and *P* > 0.001).

## Discussion

A significant difference was observed in hypercalciuria between case and control group in this study. No similar study was found since all other papers have discussed the relation between hypercalciuria and various voiding disorders, as a result no comparison might be done.

In the study of Brock, no significant difference was observed in calcium excretion between children with isolated voiding frequency and voiding frequency with dysuria (6).

Parekh et al declared that idiopathic hypercalciuria may have a specific role in urinary disorders. Although there is an impressive relation between hypercalciuria and a subgroup of urinary disorders, its mechanism is not fully understood (7).

Vachvanichsanongp and Moore showed that idiopathic hypercalciuria is related to all types of urinary incontinence and calcium to creatinine ratio of random urine which is done for hypercalciuria, is suggested to be done as a part of primary evaluation for children with urinary incontinency (8).

In the study of Fivush, the importance of idiopathic hypercalciuria evaluation of infants with dysuria and irritation, even in the absence of hematuria, was stated (9).

Yousefichaijan et al, showed effectiveness of hydrochlorothiazide, as a cheap and safe medication, to reduce recurrent abdominal pains in girls with idiopathic hypercalciuria (10).

In another study of Yousefichaijan et al in 2012, it has been hypothesized that the treatment of hypercalciuria is more useful to prevent from repetitive urinary infection and that the relation between UTI and idiopathic hypercalciuria needs more studies in which factors that are not useful have been omitted (11).

## Conclusion

The results of this study showed that there is a significant difference between case and control groups based on the presence of hypercalciuria and hypercalciuria is a cause of over active bladder. Based on the high prevalence of hypercalciuria in these patients, examination and treatment of hypercalciuria in patients with over active bladder may be effective.

## Limitations of the study

The study performed based on laboratory assay so laboratory misdiagnosis can affect our study however its effect was on each case and control group.

## Acknowledgments

There is no doubt that conduction of the present study might not be feasible without cooperation of the patients, the respected colleagues, therefore we express our high gratitude and acknowledgement to the aforementioned persons and organizations and other colleagues in this researching project.

## Authors’ contribution

All authors contributed to design of the research. PY, FD and SS conducted the research. SS and AP analyzed the data. PS and AP prepared the manuscript. All authors read, revised, and approved the final manuscript.

## Ethical considerations

Ethical issues (including plagiarism, misconduct, data fabrication, falsification, double publication or submission, redundancy) have been completely observed by the authors.

## Conflict of interests

The authors declared no competing interests.

## Funding source

This study was financially supported by Arak University of Medical Sciences (Grant# Behsan-711).
